# Instrumental Role of *Helicobacter pylori* γ-Glutamyl Transpeptidase in VacA-Dependent Vacuolation in Gastric Epithelial Cells

**DOI:** 10.1371/journal.pone.0131460

**Published:** 2015-06-25

**Authors:** Samantha Shi Min Ling, Lawrence Han Boon Khoo, Le-Ann Hwang, Khay Guan Yeoh, Bow Ho

**Affiliations:** 1 Department of Microbiology, Yong Loo Lin School of Medicine, National University of Singapore, Singapore, Singapore; 2 Monoclonal Antibody Unit, Institute of Molecular and Cell Biology, Singapore, Singapore; 3 Department of Medicine, Yong Loo Lin School of Medicine, National University of Singapore, Singapore, Singapore; Institut Pasteur Paris, FRANCE

## Abstract

*Helicobacter pylori* causes cellular vacuolation in host cells, a cytotoxic event attributed to vacuolating cytotoxin (VacA) and the presence of permeant weak bases such as ammonia. We report here the role of γ-glutamyl transpeptidase (GGT), a constitutively expressed secretory enzyme of *H*. *pylori*, in potentiating VacA-dependent vacuolation formation in *H*. *pylori*-infected AGS and primary gastric cells. The enhancement is brought about by GGT hydrolysing glutamine present in the extracellular medium, thereby releasing ammonia which accentuates the VacA-induced vacuolation. The events of vacuolation in *H*. *pylori* wild type (WT)- and Δ*ggt*-infected AGS cells were first captured and visualized by real-time phase-contrast microscopy where WT was observed to induce more vacuoles than Δ*ggt*. By using semi-quantitative neutral red uptake assay, we next showed that Δ*ggt* induced significantly less vacuolation in AGS and primary gastric epithelial cells as compared to the parental strain (*P*<0.05) indicating that GGT potentiates the vacuolating effect of VacA. Notably, vacuolation induced by WT was significantly reduced in the absence of GGT substrate, glutamine (*P*<0.05) or in the presence of a competitive GGT inhibitor, serine-borate complex. Furthermore, the vacuolating ability of Δ*ggt* was markedly restored when co-incubated with purified recombinant GGT (rGGT), although rGGT itself did not induce vacuolation independently. Similarly, the addition of exogenous ammonium chloride as a source of ammonia also rescued the ability of Δ*ggt* to induce vacuolation. Additionally, we also show that monoclonal antibodies against GGT effectively inhibited GGT activity and successfully suppressed *H*. *pylori*-induced vacuolation. Collectively, our results clearly demonstrate that generation of ammonia by GGT through glutamine hydrolysis is responsible for enhancing VacA-dependent vacuolation. Our findings provide a new perspective on GGT as an important virulence factor and a promising target in the management of *H*. *pylori*-associated gastric diseases.

## Introduction

*Helicobacter pylori* is a Gram-negative, spiral-shaped bacterium that colonizes gastric epithelial cells [1]. More than half of the world’s population has been infected with this pathogen although most remain asymptomatic [2]. Nonetheless, infection with *H*. *pylori* predisposes individuals to a spectrum of gastroduodenal diseases such as chronic gastritis, peptic ulcer disease, gastric adenocarcinoma and mucosa-associated lymphoid tissue lymphomas [1,3].

*H*. *pylori* produces several virulence factors that contribute to its pathogenesis. One of these is an enzyme, γ-glutamyl transpeptidase or GGT (EC 2.3.2.2) which is expressed in all *H*. *pylori* strains [4,5]. GGT has been implicated in various host cell damaging effects including induction of apoptosis [6], upregulation of cyclooxygenase-2 in human gastric cells [7], inhibiting T cell proliferation [8] and generating H_2_O_2_ leading to DNA damage [9]. The enzyme is first synthesized as a pro-enzyme (~60 kDa) before being subsequently processed to give a large (~37 kDa) and a small (~20 kDa) subunit. The large and small subunits then associate to form the active enzyme [4]. Biochemically, GGT catalyzes reactions in which a γ-glutamyl moiety is transferred from γ-glutamyl compounds, such as glutathione, to amino acids (transpeptidation) or water (hydrolysis).

One of the virulence processes of *H*. *pylori* is that it causes cytoplasmic vacuole formation in cells *in vitro* [10] and *in vivo* [11]. The process has been attributed to a well-studied secreted virulence factor, vacuolating cytotoxin (VacA) and the phenomenon is dependent on the presence of permeant weak bases such as ammonia [12] in the extracellular medium. A current model of the vacuolation process involves VacA being internalized into the endosomal compartment by endocytosis [13] and altering V-ATPase activity [14], thereby leading to an increased influx of H^+^ into the endosome. This then leads to an accumulation of ammonium ions and subsequent water influx into the endosomal lumen, giving rise to the osmotic swelling of late endosomes which results in the formation of massive vacuoles, both in number and size [15]. A major source of ammonia required for vacuolation is reported to come from the hydrolysis of urea by urease [16,17]. Akin to urease, GGT is also known to generate ammonia by hydrolyzing glutamine [18,19], an amino acid that is abundant in plasma [20]. Hence, it was of great interest to investigate if GGT also contributes to the vacuolation process.

To evaluate the role of GGT in the induction of cell vacuolation in gastric epithelial cells, AGS cells were co-cultured with various isogenic mutants and their vacuolating abilities were compared to that of the parental strain. Here, we provide evidence that GGT plays an equally important role, alongside VacA and urease, in the vacuolation process by generating ammonia through glutamine hydrolysis.

## Materials and Methods

### Ethics Statement

Gastric biopsies from human patients were obtained with written informed consent and the study was conducted with approval from the National Healthcare Group Domain Specific Review Board, Singapore. All procedures on the use of laboratory animals were done in accordance with the regulations and guidelines of the National Advisory Committee for Laboratory Animal Research, Singapore. The protocol was approved by the Institutional Animal Care and Use Committee of the Biological Resource Centre, Agency for Science, Technology and Research, Singapore (Protocol number #110693).

### Bacterial Strains

*H*. *pylori* 88–3887 (WT) with *vacA* s1/m1 genotype is a piglet-passaged motile variant of the non-motile strain 26695 [21] whose genome has been completely sequenced [22]. WT was routinely cultured as described [23] and was used for the construction of a *vacA*-isogenic mutant (Δ*vacA*), urease-isogenic mutant (Δ*ureAB*) and double mutants (Δ*vacA/ggt* and Δ*ureAB/ggt*) by allelic exchange [9,24]. Sequences of primers used to construct the various mutants are shown in S1 Table. The *ggt*-isogenic mutant (Δ*ggt*) had been previously constructed [9]. *Escherichia coli* Top10 and BL21(DE3) pLysS were used for amplification of plasmid DNA and expression of rGGT protein, respectively. Plasmid transformed *E*. *coli* was cultured at 37°C in Luria-Bertani medium supplemented with 50 μg/ml ampicillin.

### Construction of Recombinant Plasmid

pRSET-A expression vector (Invitrogen, Grand Island, NY) was used for the cloning of full length *ggt* excluding the signal peptide (*ggt*; 79^th^-1704^th^ bp). Purified genomic DNA from *H*. *pylori* strain 88–3887 was subjected to PCR amplification with forward primer 5’-GCCGCTGCAGCGCGAGTTACCCCCCCATTAAA-3' and reverse primer 5’-GCCGAAGCTTTTAAAATTCTTTCCTTGGATCCGTT-3’ carrying PstI and HindIII restriction sites (underlined), respectively. After digestion with the two restriction enzymes, the PCR product was then ligated into expression vector, pRSET-A at PstI and HindIII restriction sites.

### Recombinant Protein Expression and Purification

The recombinant pRSET-*ggt* was transformed into BL-21 (DE3) pLysS and induced with isopropyl-β-D-thiogalactoside (0.4mM) at 37°C for 3 hours. The expressed recombinant protein (rGGT) was purified using affinity chromatography through a Nickel chelating column (GE Healthcare, Piscataway, NJ), dialyzed against PBS (pH 7.4) overnight at 4°C and stored at -20°C until use.

### GGT Activity Assay

GGT activity was assayed according to the method described [25]. γ-glutamyl-*ρ*-nitroanilide (Sigma-Aldrich, St Louis, MO) was used as a donor substrate and glycyl-glycine (Sigma-Aldrich, St Louis, MO) was used as the glutamate acceptor for the transpeptidation reaction. Briefly, purified rGGT was incubated at 37°C for 30 minutes in 100mM Tris-HCl (pH 8.0) in the presence of 1mM γ-glutamyl-ρ-nitroanilide and 20mM glycyl-glycine. The production of free *ρ*-nitroanilide released in the incubation medium was determined by spectrophotometry at 405nm. One unit of GGT activity was defined as the quantity of enzyme that releases 1 μmol *ρ*-nitroaniline per minute per mg of protein at 37°C.

### Cell Culture and Culture Treatment Conditions

Human gastric adenocarcinoma cell line AGS (CRL-1739; American Type Culture Collection, Manassas, VA) was cultured in Ham’s F-12K medium (Sigma-Aldrich, St Louis, MO) supplemented with 10% fetal calf serum (Gibco-BRL, Rockville, MD) and 2mM glutamine at 37°C under 5% CO2. Where indicated, glutamine-free F-12K medium was also used. Primary gastric epithelial cells were isolated from human gastric biopsies and cultured as described previously [9,26]. All cells were co-cultured with 3 day-old *H*. *pylori* WT or the different isogenic mutants (Δ*ggt*, Δ*vacA*, Δ*ureAB*, Δ*vacA/ggt*, Δ*ureAB*/*ggt*) at a multiplicity of infection (MOI) of 1:100 [9]. Where indicated, cells were treated with rGGT at an approximately equivalent amount to that produced by the viable bacteria [9].

### Real-Time Phase Contrast Microscopy

AGS cells were seeded in a 35 mm plastic bottom dish (Nunc, Roskilde, Denmark) at a density of 1.5 × 10^4^ cells per cm^2^. Following 24 hours incubation at 37°C under 5% CO_2_, cells were infected with *H*. *pylori* WT or Δ*ggt*. Infected cells were then placed into the Biostation IMQ microscope chamber under 5% CO_2_ (Nikon, Tokyo, Japan) for real-time observation under phase-contrast microscopy. Uninfected AGS cells served as control. The water and chamber temperature were stabilized at 37°C to prevent focus drift during recording. Time-lapse images were taken at 5 minutes interval between each image acquisition for a total period of 24 hours. Images were viewed using ImageJ software version 1.44i.

### Assay for Cell Vacuolation

AGS cells or primary human gastric cells were seeded in a 12-well culture plate (Greiner, Frickenhausen, Germany) at a density of 1.5 × 10^4^ cells per cm^2^ and left for 24 hours as previously described [27]. After which, cells were treated with *H*. *pylori* WT or isogenic mutants for an additional 24 hours. Where indicated, rGGT (10mU), serine-borate complex (SBC), a competitive GGT inhibitor [28] (2-10mM; Sigma-Aldrich, St Louis, MO) or ammonium chloride (1-3mM) was incubated with AGS cells. At the respective time-points, cells were inspected for vacuolation using phase contrast microscopy. Extent of vacuolation was then quantified using neutral red uptake assay as previously described with slight modifications [16]. Briefly, the medium overlaying the cells was removed and replaced with 0.2 ml of freshly prepared 0.05% neutral red in PBS containing 0.3% bovine serum albumin (BSA) for 5 min. Cells were then washed thrice with 0.5 ml of PBS/0.3% BSA. The neutral red dye taken up by the vacuoles was then extracted from the cells by the addition of 0.2 ml of acidified ethanol (70% ethanol, 0.37% HCl). The optical density was measured with a microplate reader (BioRad Laboratories, Richmond, VA) at 534 nm with subtraction of absorbance at 405 nm. Neutral red dye uptake is expressed as absorbance per 10^5^ cells. All experiments were done in triplicates.

### Ammonia Assay

AGS cells were co-cultured with *H*. *pylori* WT or Δ*ggt* in the absence or presence of glutamine (2mM). After 24 hours, the ammonia concentrations in the spent cell culture medium were determined using an ammonia assay kit (BioVision, Milpitas, CA). A standard curve was generated with each assay run using ammonium chloride standard solution according to the manufacturer’s instructions. All experiments were performed in triplicates.

### Production of Monoclonal Antibodies

Monoclonal antibodies (MAbs) against rGGT were generated according to the method as previously described with slight modifications [29]. Each of the eight-week-old female BALB/c mice (n = 5) was immunized intraperitoneally with 100μg of purified rGGT emulsified in Complete Freund's adjuvant (Sigma-Aldrich, St Louis, MO). Booster injections were administered 4 times at 3-week intervals. The mouse with the highest antibody titre was selected and given a final boost (100μg of rGGT) intravenously 2 days before being sacrificed. Hybridoma cells were generated by fusing immune spleen cells with mouse SP2-0/Ag14 myeloma cells using the ClonaCell-HY hybridoma kit according to the manufacturer’s instructions (StemCell technologies, Vancouver, Canada). MAb in supernatants of hybridoma cultures were screened with an ELISA test using purified rGGT as antigen. Positive hybridoma clones were subcloned twice by limiting dilution. Ascitic fluid was obtained by introducing 10^5^ hybridoma cells intraperitoneally into BALB/c mice pre-primed with Incomplete Freund's Adjuvant (Sigma-Aldrich, St Louis, MO). All anti-GGT MAbs were purified from ascites using protein-G-Sepharose columns (GE Healthcare, Piscataway, NJ) and stored at -20°C until use. The isotype of each MAb was determined using the IsoStrip Mouse Monoclonal Antibody Isotyping Kit (Roche, Mannheim, Germany).

### Inhibition of GGT Activity by MAbs

A total of 5×10^6^ *H*. *pylori* or rGGT (5mU) was incubated with various amounts (0.25–30μg) of different MAbs for 1 hour at 37°C in a 96-well microtitre plate (0.1 ml reaction). Quantitative detection of GGT activity was carried out as previously described [4]. Percentage inhibition of GGT activity was calculated as follows:

$$\%\text{ inhibition = }\frac{\text{GGT activity without MAb}-\text{ GGT activity with MAb}}{\text{GGT activity without MAb}}\;\times \text{ 100}$$

### GGT MAbs Inhibit Vacuole Formation

In order to assess the effect of GGT MAbs on vacuolation, *H*. *pylori* WT was separately pre-incubated with the different MAbs (10μg in 0.1 ml cell culture media solution) for 1 hour at 37°C before they were used to infect AGS cells (0.7 ml final volume). Vacuolation of AGS cells was quantitated using neutral red uptake assay. Results are represented as a percentage of the maximal value of neutral red dye uptake as induced by WT:

$$\%\text{ vacuolation = }\frac{\text{Neutral red uptake of WT-infected cells in the presence of MAb}}{\text{Neutral red uptake of WT-infected cells in the absence of MAb}}\;\times \text{ 100}$$

### Statistics

Data are presented as mean ± standard deviation. All statistical tests were carried out using one-way analysis of variance and Tukey post-test. *P* values less than 0.05 were considered statistically significant.

## Results

### Purification of Recombinant *H*. *pylori* GGT

rGGT was expressed in a soluble form after induction (S1 Fig) and was eluted with 0.4mM imidazole. SDS-PAGE of His-tag purified rGGT gave large and small subunits with estimated molecular weights of 40 and 20 kDa, respectively. A 62 kDa band corresponding to the unprocessed GGT was also obtained.

### Real-Time Phase Contrast Microscopy of Vacuolation Formation in *H*. *pylori*-Infected AGS Cells

**Fig 1:**
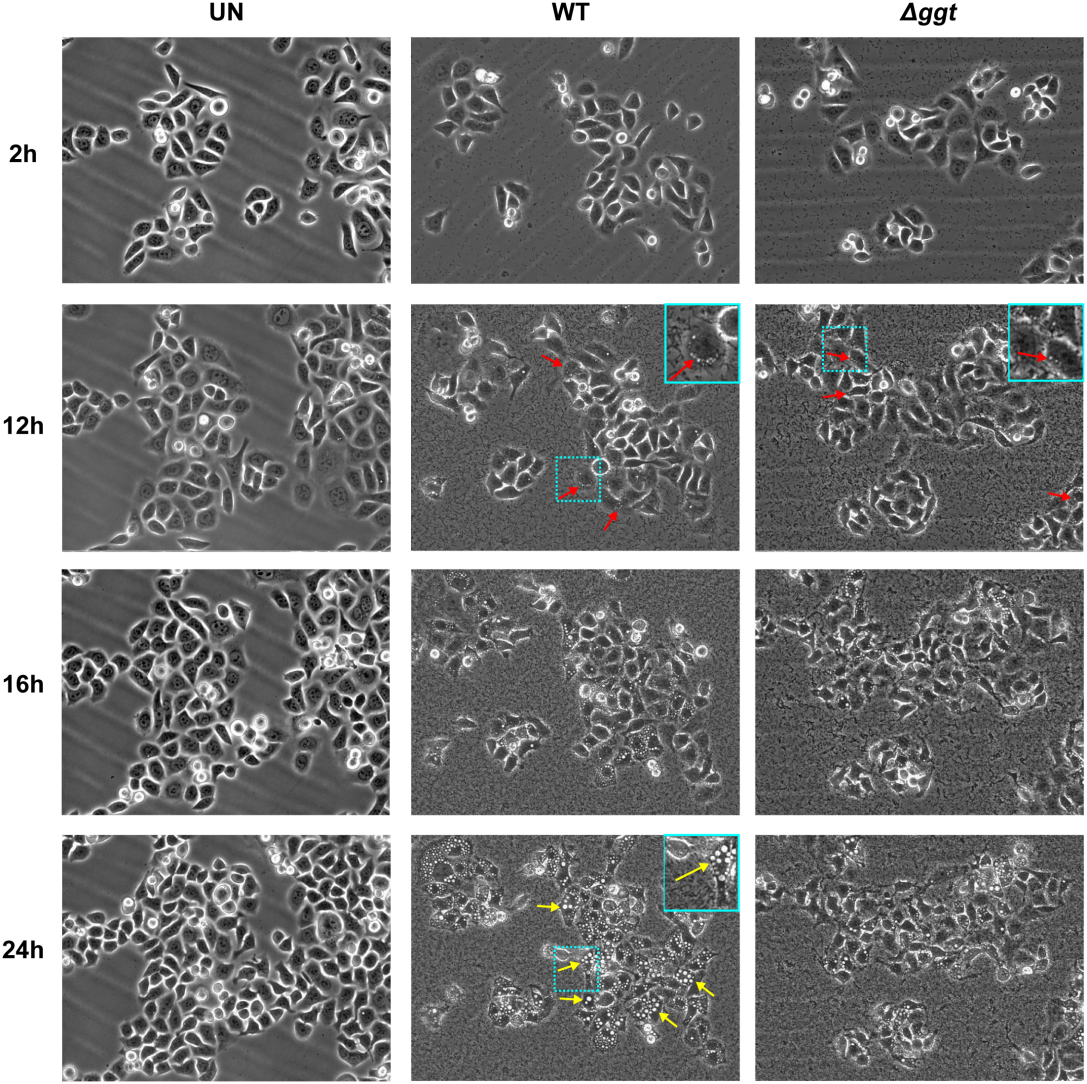
Live-cell micrographs of *H*. *pylori*-infected AGS cells. AGS cells were infected with *H*. *pylori* WT or Δ*ggt* at MOI of 1:100 over a 24 hour period and the process of infection was recorded using Biostation IMQ. Uninfected cells served as control. Micrographs shown here are at 2h, 12h, 16h and 24h time-points. Perinuclear vacuoles are indicated with red arrows. Large vacuoles are indicated with yellow arrows. Insets show a close-up of vacuole position and size in AGS cells. (See S2 Fig and S1–S3 Videos respectively for the full time course).

Live cell imaging of infected AGS cells was carried out to compare if WT and Δ*ggt* induced vacuolation in cells. Uninfected cells served as control. As observed in Fig 1, micrographs of selected time points showing cell morphology of AGS cells were fairly similar when infected with either WT or Δ*ggt* for the first 12 hours where both strains induced the formation of small vacuoles at the perinuclear region of AGS cells (red arrows). The number and size of vacuoles continued to increase until about 16 hours post-infection where more apparent differences then began to appear. In particular, in WT-infected AGS cells, the number and size of vacuoles continued to increase until the end of the recording at 24h (yellow arrows). In contrast, in the Δ*ggt*-infected cells, the number and size of vacuoles were observed to have plateaued and did not show any marked increase after 16 hours. Minimal vacuolation was observed in uninfected cells up to 24 hours. Micrographs of infected AGS cells at 2h-intervals over a 24h period are shown in S2 Fig.

### Effect of GGT and VacA on Vacuolation in AGS and Primary Gastric Epithelial Cells

**Fig 2:**
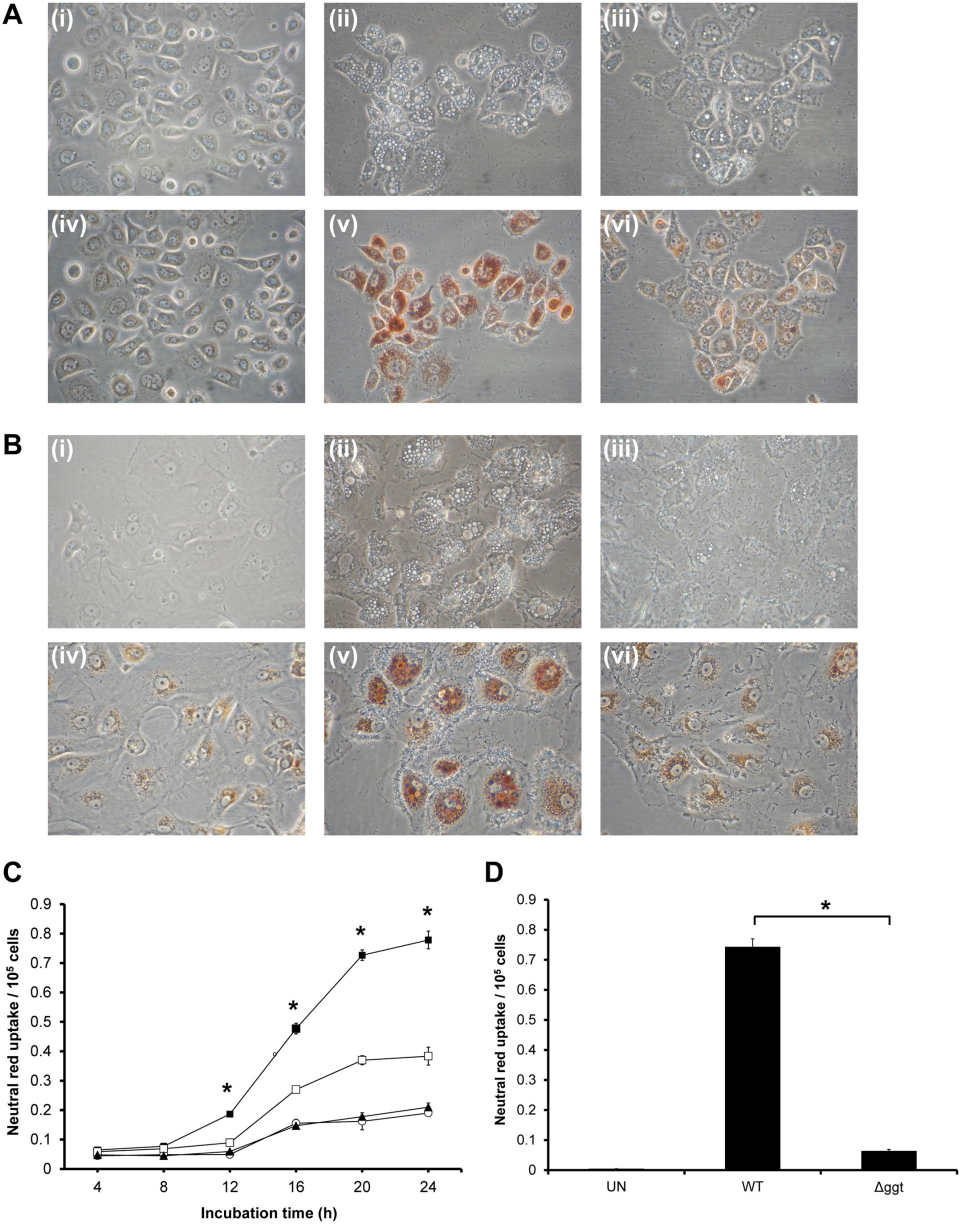
GGT contributes to vacuolation induced by *H*. *pylori*. Phase-contrast micrographs of (A) AGS cells and (B) primary gastric epithelial cells before and after neutral red uptake assay following co-culturing at MOI of 1:100 with *H*. *pylori* WT or Δ*ggt* for 24 hours. Uninfected cells (i), WT-treated cells (ii) or Δ*ggt*-treated cells (iii) were observed using phase contrast microscopy. The cells were subjected to neutral red dye assay (iv, v and vi, respectively) and observed under the microscope. Original magnification, ×400. Extent of vacuolation is indicated by the amount of neutral red retained within the cells. (C) AGS cells were co-cultured with WT *H*. *pylori* (■) or Δ*ggt* (□) at MOI 100 for various time-points up to 24 hours. (▲) indicates uninfected cells. The cells were then subjected to neutral red dye uptake assay. (D) Primary gastric epithelial cells were co-cultured with *H*. *pylori* WT or Δ*ggt* for 24 hours and subjected to neutral red uptake assay. Experiments were performed in triplicates and values represent the means ± SD from 3 independent experiments. *P<0.05.

To further investigate if GGT played a role in vacuolation formation in host gastric cells, AGS cells and primary gastric epithelial cells were co-cultured with WT or Δ*ggt*. Under phase-contrast microscopy, visible vacuolation and neutral red dye uptake were observed at 24 hours post-infection in WT-infected cells while vacuolation was shown to be markedly reduced in Δ*ggt*-treated cells (Fig 2A and 2B). This finding is in agreement with the real-time microscopy observations. This was further confirmed by a semi-quantitative assay based on neutral red uptake where AGS cells co-cultured with Δ*ggt* accumulated significantly reduced neutral red dye uptake than cells co-cultured with the parental strain (*P*<0.05) (Fig 2C). Primary gastric epithelial cells subjected to the same procedures showed a similar pattern (Fig 2D).

**Fig 3:**
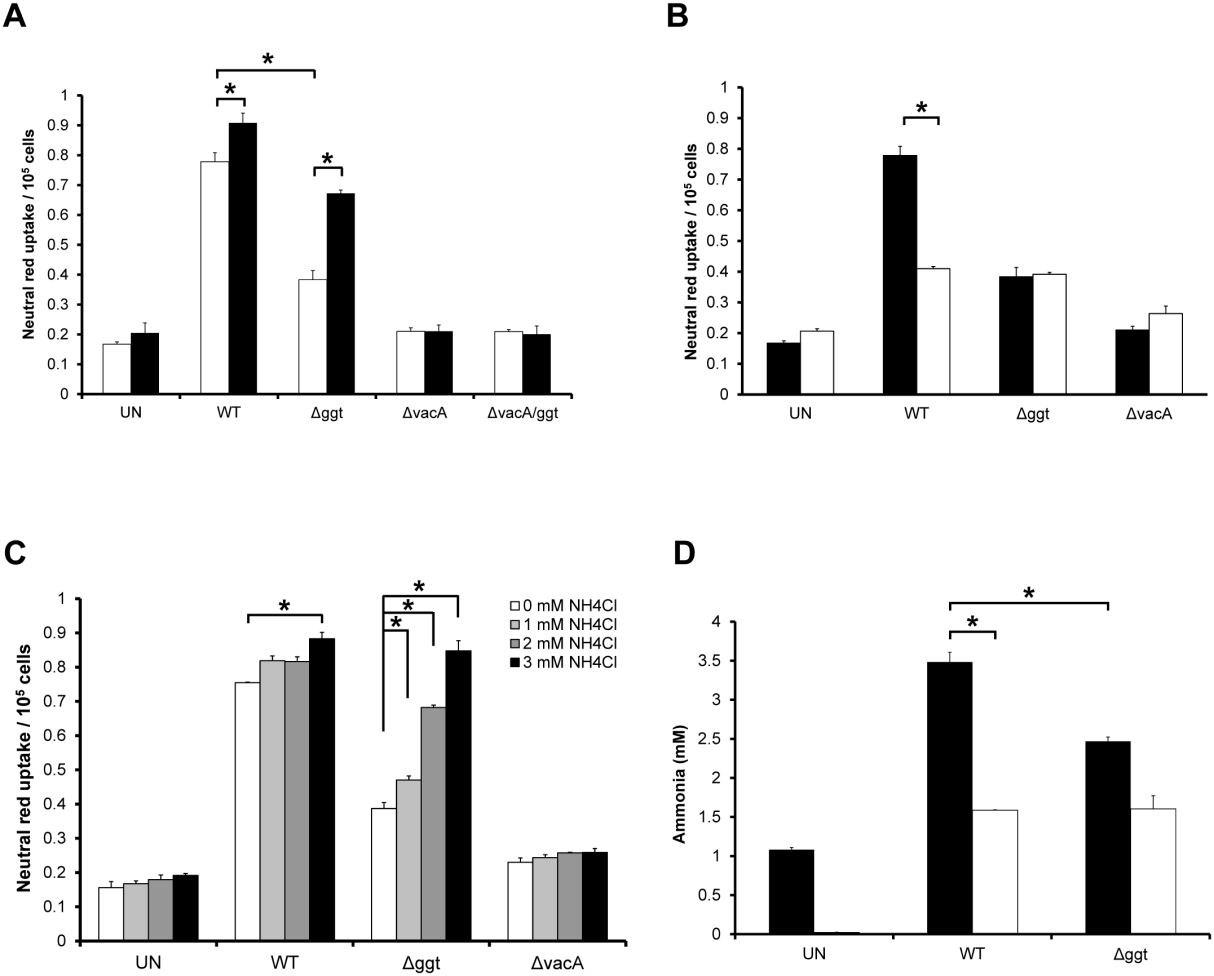
*H*. *pylori* GGT potentiates VacA-dependent vacuolation induction in gastric epithelial cells through glutamine hydrolysis. (A) AGS cells were co-cultured with *H*. *pylori* WT, Δ*ggt*, Δ*vacA*, Δ*vacA/ggt* (□) and/or rGGT (■) for 24 hours. Untreated cells (UN) served as control. (B) AGS cells were co-cultured with WT, Δ*ggt* or Δ*vacA* at MOI 1:100 for 24 hours in the presence (■) or absence (□) of 2mM glutamine. (C) AGS cells were co-cultured with WT, Δ*ggt* or Δ*vacA* at MOI 1:100 for 24 hours in the absence or presence of 1-3mM ammonium chloride. The cells were then subjected to neutral red dye uptake assay. (D) AGS cells were co-cultured with *H*. *pylori* WT or Δ*ggt* for 24 hours in the presence (■) of 2mM glutamine or in glutamine-free media (□). Concentration of ammonia in the spent culture media was measured using an ammonia assay kit. Experiments were performed in triplicates and values represent the means ± SD from 3 independent experiments. *P<0.05.

With VacA being the inducer of vacuolation in host cells as reported earlier [30], its involvement was also examined using neutral red uptake assay after co-culturing AGS cells with Δ*vacA* as well as Δ*vacA/ggt*. The results show that both mutants Δ*vacA* and Δ*vacA/ggt* induced minimal vacuolation in AGS cells even after 24 hours post-infection (Fig 3A), indicating that the vacuolation observed is dependent on the presence of VacA.

### rGGT Potentiates Vacuolation

AGS cells were treated with rGGT to determine if GGT alone could induce vacuolation in the cells. There was no significant difference (*P*>0.05) in neutral red dye uptake between rGGT-treated and untreated cells after 24 hours (Fig 3A), implying that rGGT does not possess intrinsic vacuolating activity. This was confirmed when both Δ*vacA* and Δ*vacA/ggt* induced minimal levels of neutral red uptake. Interestingly, when rGGT was supplemented into Δ*ggt-*treated AGS culture for 24 hours, vacuolation was restored to almost a similar level as that induced by WT (*P*<0.05), an effect not seen when rGGT was supplemented to Δ*vacA*- or Δ*vacA/ggt*-infected cells. The results indicate that GGT is indeed unable to induce vacuolation in the absence of VacA but is involved in exacerbating its effect. It was therefore not surprising that the co-incubation of WT and rGGT also significantly increased the extent of vacuolation compared to that induced by WT alone (*P*<0.05), reiterating the potentiating effect of GGT on VacA in vacuolation (Fig 3A).

### Role of Glutamine in the Vacuolation Process

Glutamine, being one of the substrates of GGT, generates ammonia upon hydrolysis by GGT. As vacuolation depends not only on VacA, but also on the presence of permeant weak bases such as ammonia [12], investigation was performed to determine if the absence of glutamine in the medium would affect the extent of vacuolation in *H*. *pylori*-infected AGS cells. It was observed that there was a significant decrease (*P*<0.05) in neutral red dye uptake in WT-infected cells in the absence of glutamine (Fig 3B). In comparison, neutral red dye uptake by AGS cells co-cultured with Δ*ggt* or Δ*vacA* were not affected in glutamine-free medium. As a precaution, cell viability of cells cultured with or without 2mM glutamine for 24h was also measured and no significant differences were observed (S3 Fig). Taken together, the results indicate that glutamine could be a potential source of ammonia which is vital in the vacuolation process.

## Effect of Addition of Ammonium Chloride

We next sought to determine if the addition of an external source of ammonia could restore the vacuolating effect of Δ*ggt* on AGS cells. In these experiments, WT or Δ*ggt* was co-cultured with AGS cells in the presence of 1-3mM ammonium chloride. A maximum concentration of 3mM ammonium chloride was chosen as higher concentrations of ammonium chloride (≥ 5mM) have been found to induce vacuolation independently of VacA [12]. Interestingly, exogenous addition of ammonium chloride restored vacuolation induced by Δ*ggt* in a dose-dependent manner and was fully restored to the level of that induced by the parental strain in the presence of 3mM ammonium chloride (Fig 3C). In contrast, neutral red uptake by uninfected cells or AGS co-cultured with Δ*vacA* did not show significant difference in neutral red uptake, confirming that 3mM ammonium chloride does not induce vacuolation intrinsically. These results suggest that the absence of GGT in Δ*ggt* affects its ability in inducing vacuolation in AGS cells due to a decrease in ammonia production by the isogenic mutant. This hypothesis was further strengthened by measuring the concentration of ammonia in the cell culture medium of WT and Δ*ggt*-infected AGS cells (Fig 3D). The results show that concentration of ammonia in the cell culture medium of AGS cells infected with WT was significantly higher than Δ*ggt* in the presence of 2mM glutamine (*P*<0.05). In contrast, no significant differences were observed between them in glutamine-free media.

### Vacuolation Induced by Urease-Isogenic and Double Mutants

**Fig 4:**
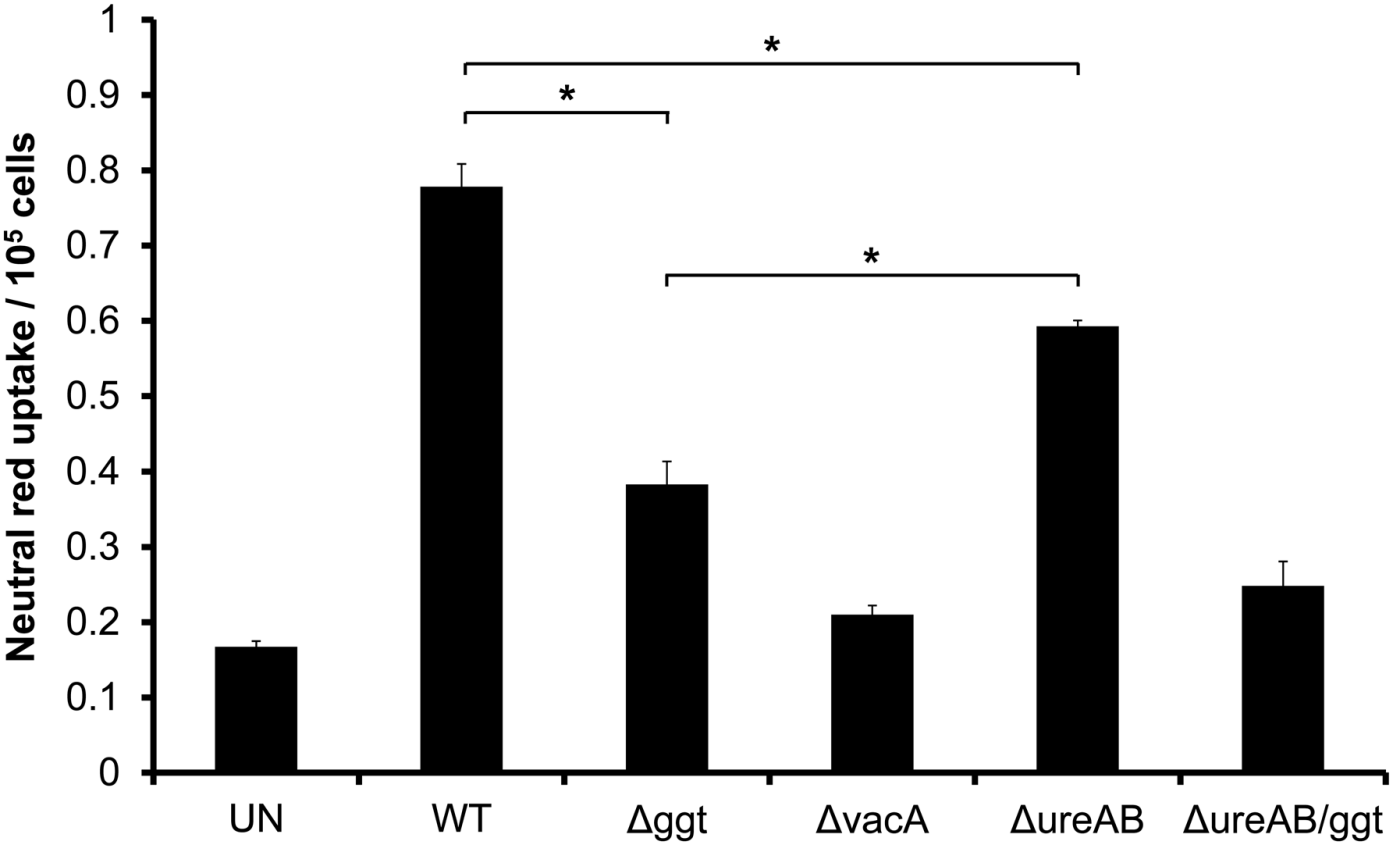
Role of GGT and urease in VacA-mediated vacuolation. AGS cells were co-cultured with WT, Δ*ggt*, Δ*vacA*, Δ*ureAB* or Δ*ureAB/ggt* at MOI 1:100 for 24 hours. Vacuolating activity was measured using neutral red dye uptake assay. Experiments were performed in triplicates and values represent the means ± SD from 3 independent experiments. *P<0.05.

Urease has been reported to augment vacuolation induced by VacA in HeLa cells via metabolism of urea to carbon dioxide and ammonia [16]. To investigate the importance of urease and GGT in vacuolation induction in AGS cells, we compared the neutral red uptake by AGS cells co-incubated with Δ*ggt* or Δ*ureAB* (Fig 4). It was observed that both Δ*ggt* and Δ*ureAB* induced significantly less vacuolation as compared to WT (*P*<0.05). Interestingly, induction of vacuolation by Δ*ggt* was significantly less than that induced by Δ*ureAB* (*P*<0.05). In addition, the double mutant, Δ*ureAB/ggt* abolished vacuolation induction to a level similar to that of Δ*vacA*. Taken together, the results show that GGT and urease are the two main potentiators of VacA in relation to vacuolation.

### Characterization of MAbs Against rGGT

Ten hybridomas were selected following the fusion of mouse myeloma cells with B lymphoblasts from one of the mice immunized with purified rGGT. All of the MAbs produced were of the IgG class and could be classified into three isotypes, IgG_l_, IgG_2a_ and IgG_2b_. S4 Fig shows the results of Western blots of SDS-PAGE gels with MAbs, as well as a polyclonal anti-*H*. *pylori* GGT antibody. Six of the MAbs recognized only the large subunit while the other four recognized only the small subunit. Results are summarized in S2 Table.

### Neutralization of GGT Activity by MAbs

**Fig 5:**
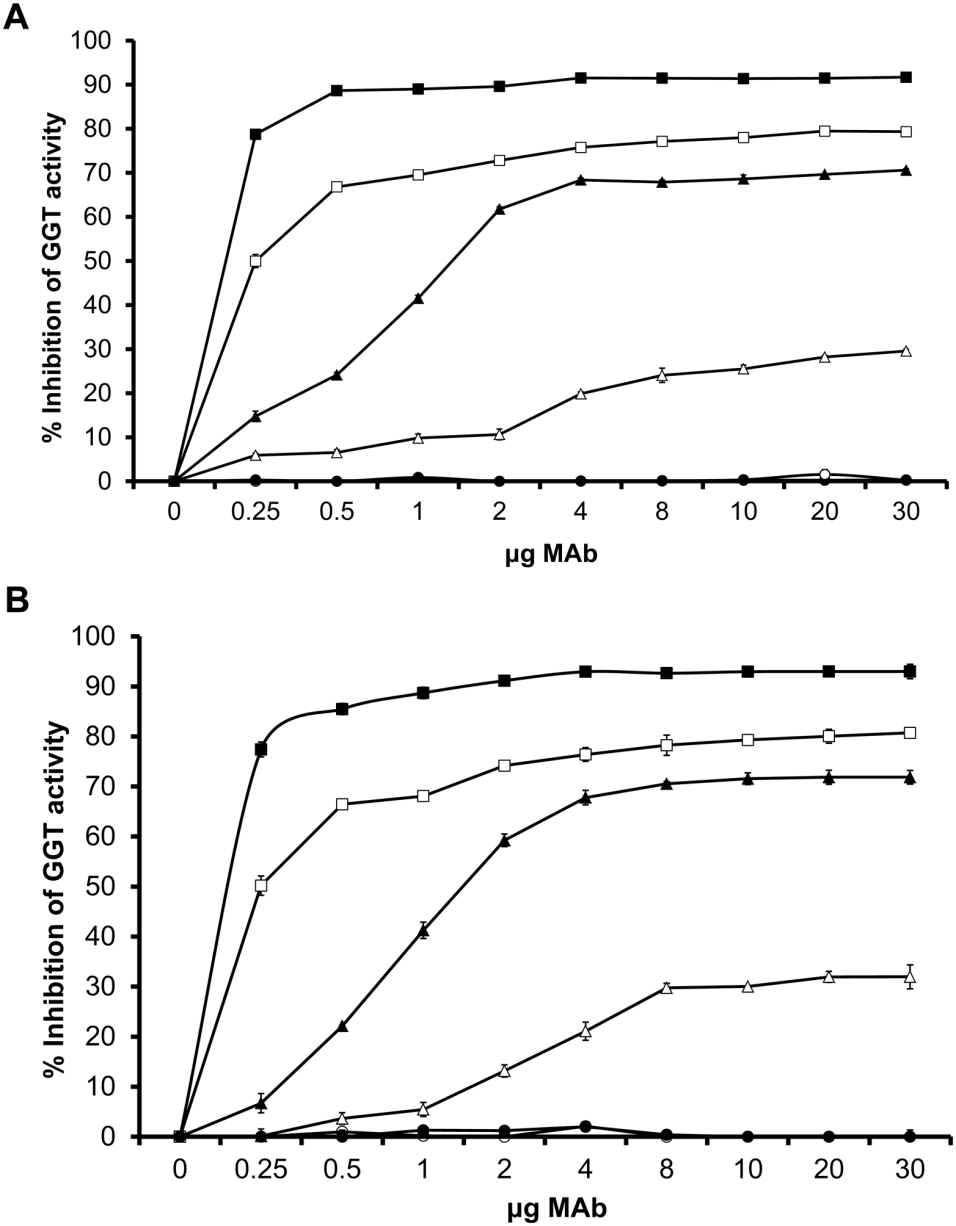
Neutralizing ability of GGT MAbs on rGGT and *H*. *pylori* 88–3887 GGT activity. (A) rGGT (5mU) or (B) a population of 5 × 10^6^ *H*. *pylori* WT was pre-incubated with various amounts of MAb 1G1 (■), 4F11 (□), 3F4 (▲) or 3C10 (Δ) (0 to 30μg of IgG protein) for 1 hour at 37°C on microtitre plates. Mouse IgG_2a_ (●) and IgG_2b_ (○) isotype controls were used in parallel at the same concentrations. Inhibition of GGT activity by MAbs was measured using GGT assay and values represent GGT activity inhibition (expressed as a percentage) compared to the control where no MAb was added. Experiments were performed in triplicates and values represent the means ± SD from 3 independent experiments.

Among the 10 MAbs, four MAbs (1G1, 3C10, 3F4 and 4F11) showed varying degrees of ability (ranging from ~30 to ~93%) in inhibiting the GGT activities of both rGGT as well as *H*. *pylori* (Fig 5A and 5B respectively). All four MAbs recognized the small subunit of GGT. The most efficient inhibitor 1G1, inhibited 77% of *H*. *pylori* WT GGT activity even at a low dose of 0.25μg and achieved maximum inhibition of 93% at 4μg. MAbs 4F11, 3F4 and 3C10 inhibited 81%, 72% and 32% *H*. *pylori* GGT activity respectively when the maximum dose of 30μg was used. The other six MAbs specific for the large subunit failed to inhibit *H*. *pylori* GGT activity even at the highest dose tested. In addition, mouse IgG_2a_ and IgG_2b_ isotype negative controls did not inhibit GGT activity.

### Inhibition of Vacuolation by MAbs and SBC

**Fig 6:**
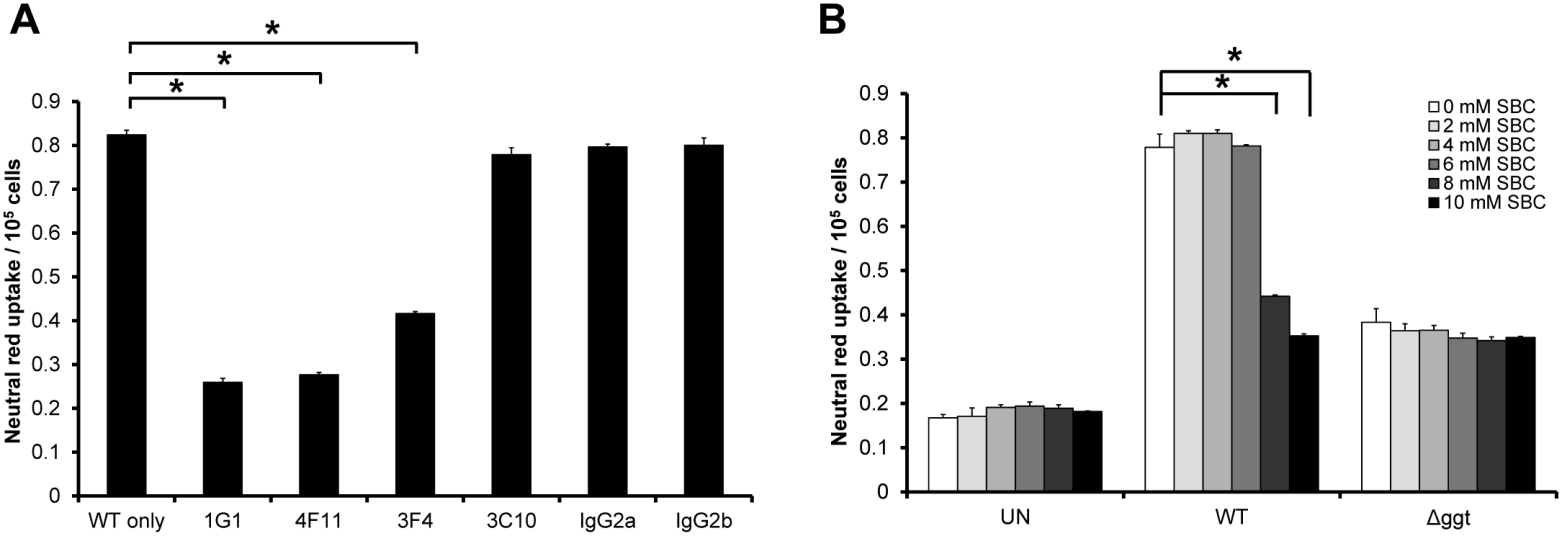
Inhibition of GGT activity decreases *H*. *pylori*-induced vacuolation. (A) *H*. *pylori* WT was pre-incubated with GGT MAbs (10μg) for 1 hour before they were used to infect AGS cells for another 24 hours. Mouse IgG_2a_ and IgG_2b_ isotype controls were used in parallel at the same concentration. (B) AGS cells were co-cultured with *H*. *pylori* WT or Δ*ggt* in the absence or presence of SBC (2-10mM). Uninfected cells (UN) served as control. The cells were then subjected to neutral red dye uptake assay. Experiments were performed in triplicates and values represent the means ± SD from 3 independent experiments. *P<0.05.

We next sought to investigate if the four MAbs which were able to inhibit GGT activity could also neutralize vacuolation induction in *H*. *pylori-*infected AGS cells. Of the four MAbs, three of them (1G1, 4F11 and 3F4) significantly inhibited vacuolation (*P*<0.05) as shown in Fig 6A. Their ability to inhibit vacuolation generally coincided with their potency in neutralizing GGT activity. As a control, mouse IgG_2a_ and IgG_2b_ isotype negative controls failed to inhibit vacuolation. Similarly, in the presence of SBC, a competitive GGT inhibitor [28], vacuolation induction by WT in AGS cells was significantly decreased (*P*<0.05) while the level of vacuolation remained unchanged in Δ*ggt*-treated cells (Fig 6B). The effect of SBC in inhibiting vacuolation was also observed to be dose-dependent but required a minimum concentration of 8mM SBC to be effective. This further indicates that vacuolation induced by *H*. *pylori* is greatly dependent on GGT activity.

## Discussion

GGT plays multiple roles in the pathogenesis of *H*. *pylori* infections [9], having been proposed to be an essential colonizing factor in mice [4] and to induce apoptosis of host cells [6]. We also observed that cell viability of AGS cells infected with WT was significantly lower than those infected with Δ*ggt* 24 hours post-infection (S5 Fig). In addition to the numerous reported functions of GGT, we present evidence of another important role of GGT, particularly in VacA-dependent vacuolation. In this study, vacuolation formation as measured by neutral red assay showed that AGS cells co-cultured with Δ*ggt* displayed >2-fold lower (*P*<0.05) neutral red dye uptake as compared to WT at 24 hours post-infection (Fig 2C), a phenomenon also observed when using primary gastric cells (Fig 2D). Interestingly, a similar trend was also observed when we compared vacuolation induced by 5 other *H*. *pylori* strains and their respective *ggt*-isogenic mutants on both AGS and MKN28 cell lines (S6 Fig), indicating that potentiation of vacuolation by GGT is not restricted to a particular strain nor cell line only.

Previous studies have shown that VacA induces the formation of vacuoles in cells but not unless ammonia or other permeant weak bases are present in the extracellular medium [12,31]. With the use of various isogenic mutants, purified rGGT and a series of media modifications, we show in this study that GGT enhances vacuolation by hydrolyzing glutamine present in the extracellular medium to release ammonia which accentuates VacA-induced vacuolation. As a precaution, we also show that VacA expression was not affected by the deletion of the *ggt* gene (S7 Fig). Furthermore, this study has demonstrated that neutralizing monoclonal antibodies raised against rGGT as well as the chemical GGT inhibitor, SBC, are successful in inhibiting vacuolation.

It has been previously reported that urease is the main potentiator of VacA-dependent vacuolation by converting urea to carbon dioxide and ammonia [16]. However, our results indicate that it is not the only factor contributing to the generation of ammonia required for vacuolation as Δ*ureAB* was still able to induce a significant amount (*P*<0.05) of vacuolation in AGS cells (Fig 4). This agrees with a previous report whereby vacuolation induced in Sfl Ep cells by broth-culture filtrates from a urease-isogenic mutant did not differ significantly to that induced by the parental strain in the absence of urea [32]. In the same report, it was also found that a large portion of ammonia in the broth filtrates was a urease-independent metabolic product of the bacteria, indicating that there are other bacterial factors responsible for the production of ammonia essential for vacuolation induction. In this study, we show that neutral red uptake induced by Δ*ggt* was significantly lesser than that induced by Δ*ureAB* (where GGT is still present), emphasizing that GGT is the other important ammonia-generating factor alongside urease under the tested culture conditions. Taken together, the results from this study strongly affirm that although GGT does not have intrinsic vacuolating activity, it plays an important role alongside VacA and urease in inducing vacuolation in gastric epithelial cells.

Induction of cell vacuolation is strongly associated with pathogenic strains of *H*. *pylori* and is believed to induce cell structural damage, cell suffering and eventually cell death [33], resulting in a chronic inflammatory response in the gastric mucosa of the host [27]. In this study, we have shown that *H*. *pylori* GGT plays an important role in contributing to VacA-induced vacuolation. Vacuolation in biopsies from *H*. *pylori*-infected individuals has been frequently reported by many groups [34–38]. Importantly, we have also shown in this study that prominent vacuolation was observed in primary gastric epithelial cells upon *H*. *pylori* infection, indicating that *H*. *pylori* is able to induce vacuolation in these cells. However, it is noted that the extent of vacuolation in vivo may not be as prominent as that observed in vitro which may be due to several factors such as differences in concentrations of VacA in vivo and in the detectability of it in epithelial cells contained within an intact mucosal surface as opposed to flattened, adherent cells of a monolayer [39].

In conclusion, this work has revealed another important role of GGT, particularly in accentuating VacA-dependent vacuolation. This is a novel pathogenic effect of GGT which has hitherto not been reported and hence could be another reason as to why *H*. *pylori* with higher GGT activity is associated with more severe gastroduodenal diseases as was previously reported [9]. The fact that *ggt* gene is present in all *H*. *pylori* isolates [4,5,9] further illustrates the vital role of this enzyme in potentiating virulence. Intriguingly, it has been reported that other VacA activities such as induction of apoptosis and epithelial permeability can be potentiated by ammonia [17,40], thus raising the possibility that these activities may also be potentiated by GGT-produced ammonia and would certainly be an interesting area to explore. Taken together, our findings provide a new perspective on GGT as a potent virulence factor and a potential target in the management of *H*. *pylori*-associated infections.

## Supporting Information

S1 Fig Purification of rGGT. Lane M, Prestained Precision Protein Standards (BioRad Laboratories); Lane 1, Soluble fraction of sonicated cells; Lanes 2–3, Flow-through fractions; Lanes 4–5, Eluted fractions from His-tag column.
(PDF)

S2 Fig Time-lapse micrographs of *H*. *pylori*-infected AGS cells. AGS cells were infected with (A) *H*. *pylori* WT or (B) Δ*ggt* at MOI of 1:100 over a 24 hour period. (C) Uninfected cells served as control. Time-lapse micrographs are shown at 2 hourly intervals. Time from the start of infection is indicated in white. Scale bar represents 50 μm. (See S1, S2 and S3 Videos respectively for the full time course).
(PDF)

S3 Fig Cell viability of AGS cells in the presence or absence of glutamine. AGS cells were cultured in glutamine-free medium or medium containing 2mM glutamine for 24 hours. Cell viability was measured by MTT assay.
(PDF)

S4 Fig Specificity of MAbs raised against rGGT. Western blot of SDS-PAGE gels of *H*. *pylon* 88–3887 lysate with 10 MAbs and polyclonal anti-*H*. *pylon* GGT antibody. Lanes: 1, 1G5; 2, 1G10; 3, 1H5; 4, 2B5; 5, 2G1; 6, 4A11; 7, 1G1; 8, 3C10; 9, 3F4; 10, 4F11; 11, polyclonal anti-*H*. *pylori* GGT mouse IgG. Molecular weight markers are shown on the left.
(PDF)

S5 Fig Cell viability of AGS cells after co-culture with *H*. *pylori* or rGGT. AGS cells were incubated with *H*. *pylori* WT, *ggt*-isogenic mutant or rGGT for 24 hours. Uninfected cells (UN) were included as the control. Results are represented as fold difference with respect to uninfected cells (taken as 1). *P<0.05.
(PDF)

S6 Fig Potentiation of vacuolation by *H*. *pylori* GGT is not strain-dependent nor cell-line dependent. (A) AGS and (B) MKN28 cells were co-cultured with various *H*. *pylori* strains and their respective *ggt*-isogenic mutants as indicated for 24 hours (MOI 1:100). Two standard *H*. *pylori* strains (88–3887 and 26695) and four clinical strains (789, 840, 1034, 1018 were tested. The cells were subjected to neutral red uptake assay. Uninfected cells (UN) served as control. The clinical strains are all Type I strains with *vacA* s1 genotype and were isolated from gastric biopsies obtained from the gastric antrum within 2 cm of the pylorus in patients who underwent upper gastrointestinal endoscopy at the National University Hospital, Singapore. Strains 789 and 1018 were isolated from patients with gastric ulcer while strains 840 and 1034 were isolated from patients with non-ulcer dyspepsia. Experiments were performed in triplicates and values represent the means ± SD from 3 independent experiments. *P<0.05.
(PDF)

S7 Fig Deletion of *ggt* gene does not affect the expression of VacA. Western blot analysis of *H*. *pylori* lysates probed using antibody against VacA. Lane 1, *H*. *pylori* WT lysate; Lane 2, Δ*ggt* lysate; Lane 3, Δ*vacA* lysate.
(PDF)

S1 Table Primers used for construction of various mutants of *H*. *pylori*. (PDF)

S2 Table Summary of MAb isotypes and specificities. (PDF)

S1 Video Live-cell imaging of *H*. *pylori* WT-infected AGS cells. (WMV)

S2 Video Live-cell imaging of *H*. *pylori* Δ*ggt*-infected AGS cells. (WMV)

S3 Video Live-cell imaging of uninfected AGS cells. (WMV)
