# Supplementary material for: Instrumental Role of Helicobacter pylori γ-Glutamyl Transpeptidase in VacA-Dependent Vacuolation in Gastric Epithelial Cells
Source: PLoS One. 2015 Jun 25;10(6):e0131460. doi: 10.1371/journal.pone.0131460 (PMC4482420; doi:10.1371/journal.pone.0131460)
Supplement: S1 Fig — Lane M, Prestained Precision Protein Standards (BioRad Laboratories); Lane 1, Soluble fraction of sonicated cells; Lanes 2–3, Flow-through fractions; Lanes 4–5, Eluted fractions from His-tag column. (PDF) [file pone.0131460.s001.pdf]

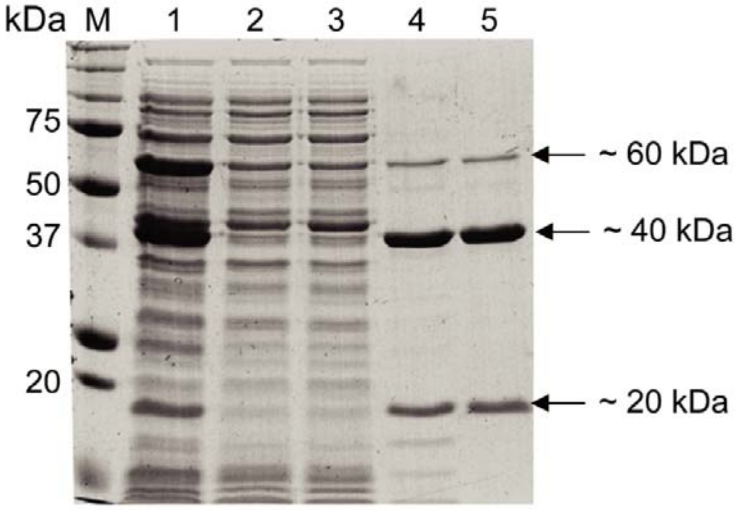


**S1 Figure. Purification of rGGT**. Lane M, Prestained Precision Protein Standards (BioRad Laboratories); Lane 1, Soluble fraction of sonicated cells; Lanes 2-3, Flow-through fractions; Lanes 4-5, Eluted fractions from His-tag column.
