# Supplementary material for: Instrumental Role of Helicobacter pylori γ-Glutamyl Transpeptidase in VacA-Dependent Vacuolation in Gastric Epithelial Cells
Source: PLoS One. 2015 Jun 25;10(6):e0131460. doi: 10.1371/journal.pone.0131460 (PMC4482420; doi:10.1371/journal.pone.0131460)
Supplement: S2 Fig — AGS cells were infected with (A) H. pylori WT or (B) Δggt at MOI of 1:100 over a 24 hour period. (C) Uninfected cells served as control. Time-lapse micrographs are shown at 2 hourly intervals. Time from the start of infection is indicated in white. Scale bar represents 50 μm. (See S1, S2 and S3 Videos respectively for the full time course). (PDF) [file pone.0131460.s002.pdf]

(A)

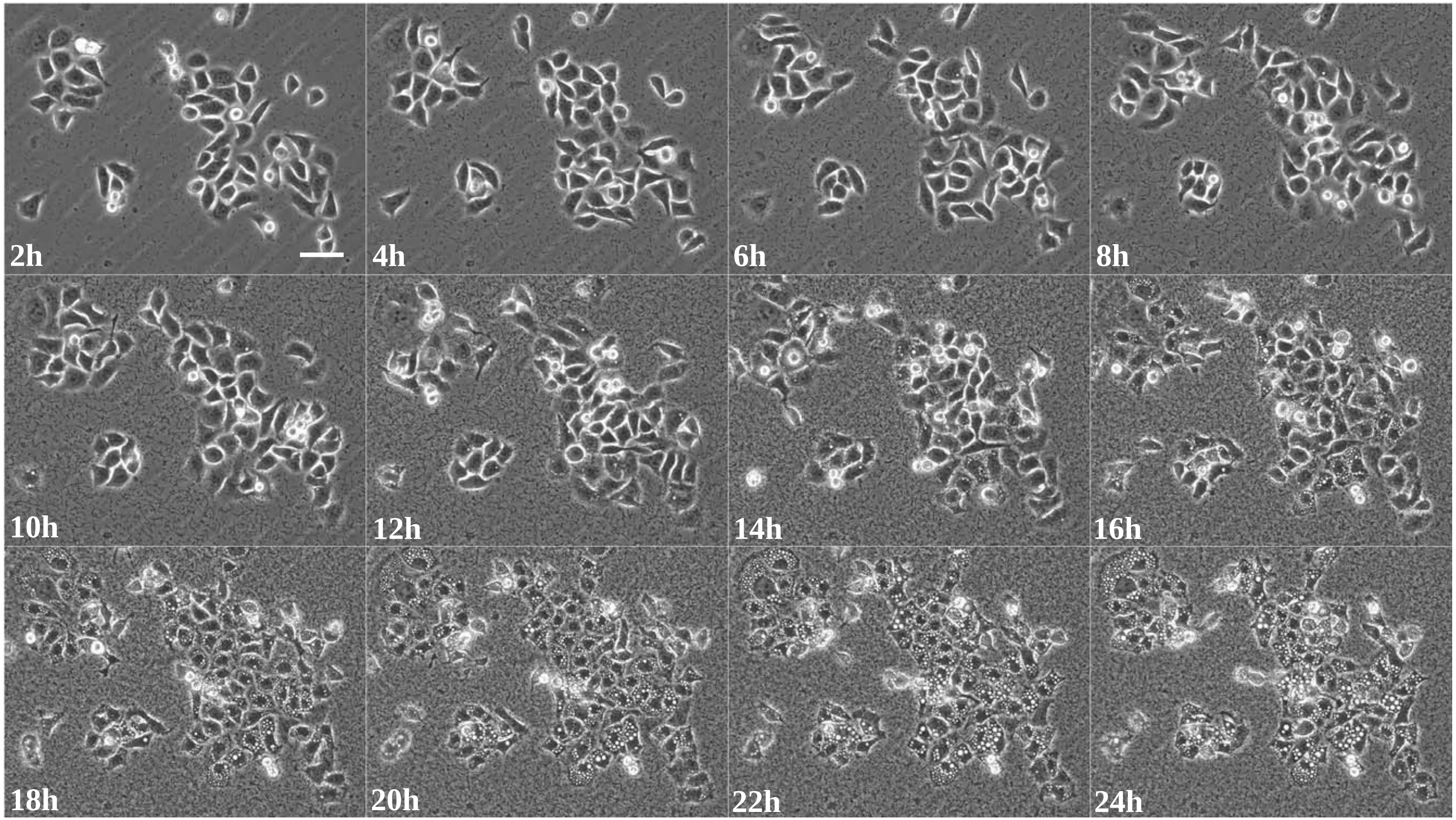

(B)

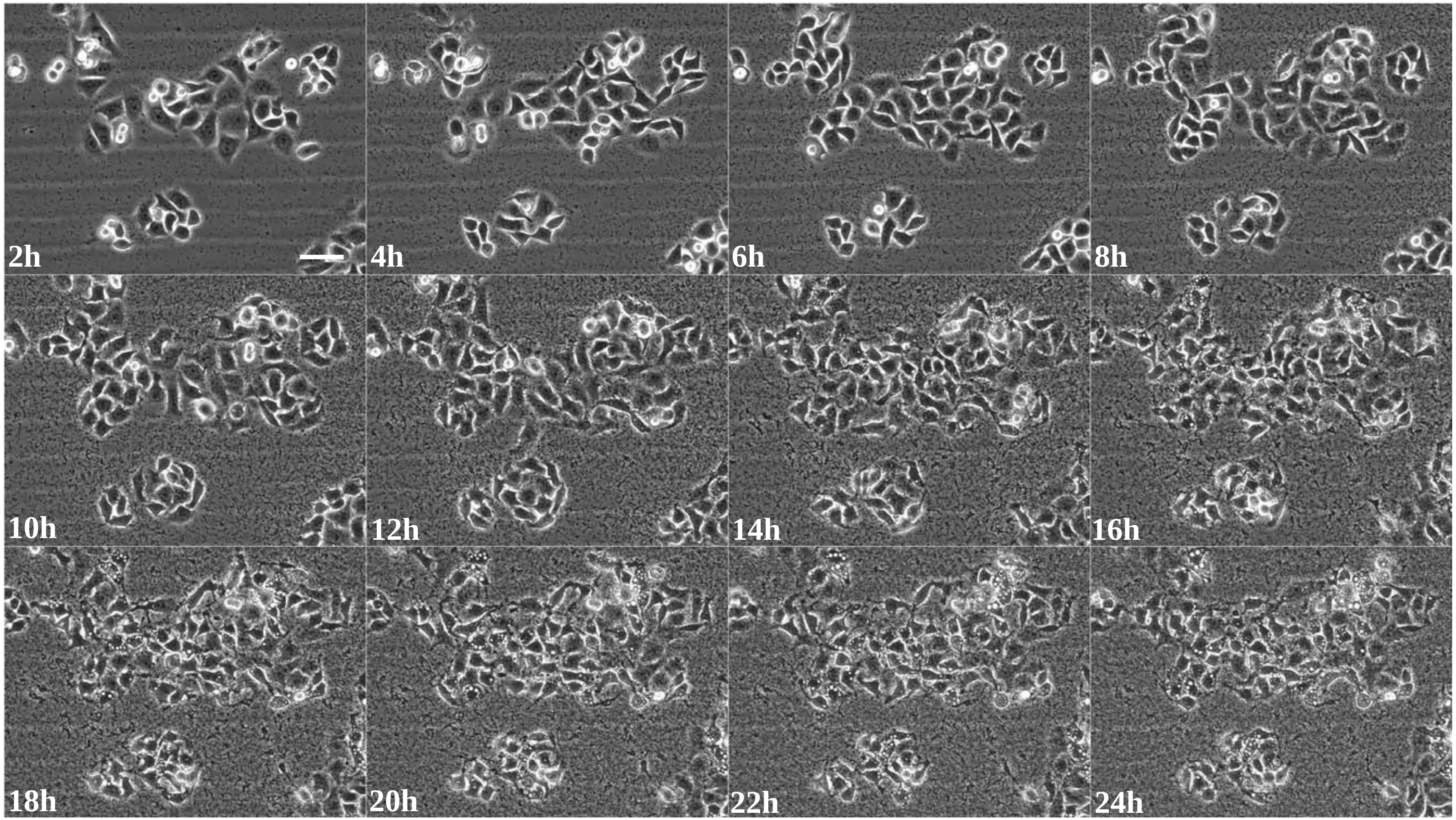

(C)

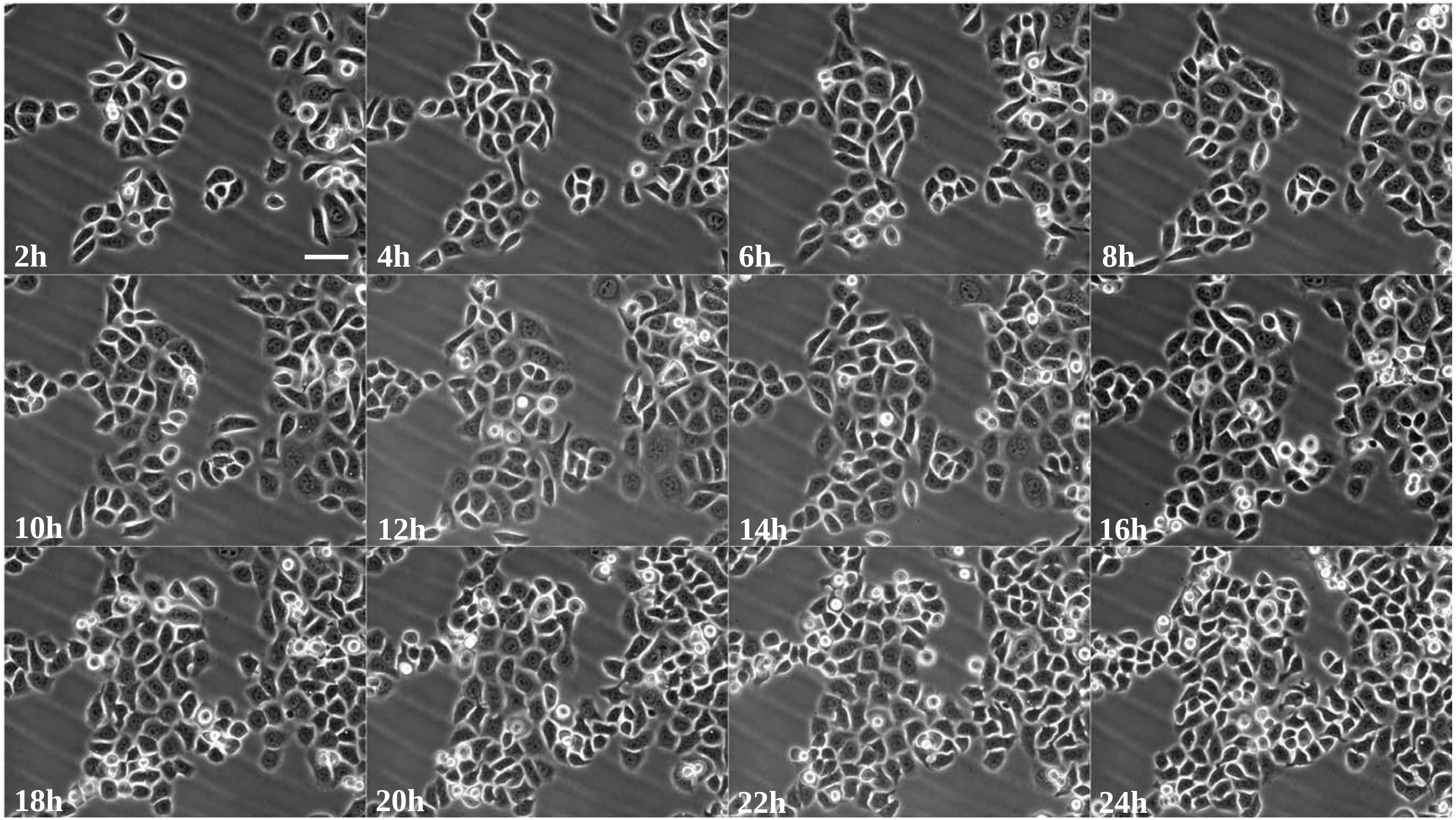

S2 Figure. Time-lapse micrographs of *H. pylori*-infected AGS cells. AGS cells were infected with (A) *H. pylori* WT or (B) Δ*ggt* at MOI of 1:100 over a 24 hour period. (C) Uninfected cells served as control. Time-lapse micrographs are shown at 2 hourly intervals. Time from the start of infection is indicated in white. Scale bar represents 50 µm. (See S1, S2 and S3 Videos respectively for the full time course).
