# Supplementary material for: Instrumental Role of Helicobacter pylori γ-Glutamyl Transpeptidase in VacA-Dependent Vacuolation in Gastric Epithelial Cells
Source: PLoS One. 2015 Jun 25;10(6):e0131460. doi: 10.1371/journal.pone.0131460 (PMC4482420; doi:10.1371/journal.pone.0131460)
Supplement: S3 Fig — AGS cells were cultured in glutamine-free medium or medium containing 2mM glutamine for 24 hours. Cell viability was measured by MTT assay. (PDF) [file pone.0131460.s003.pdf]

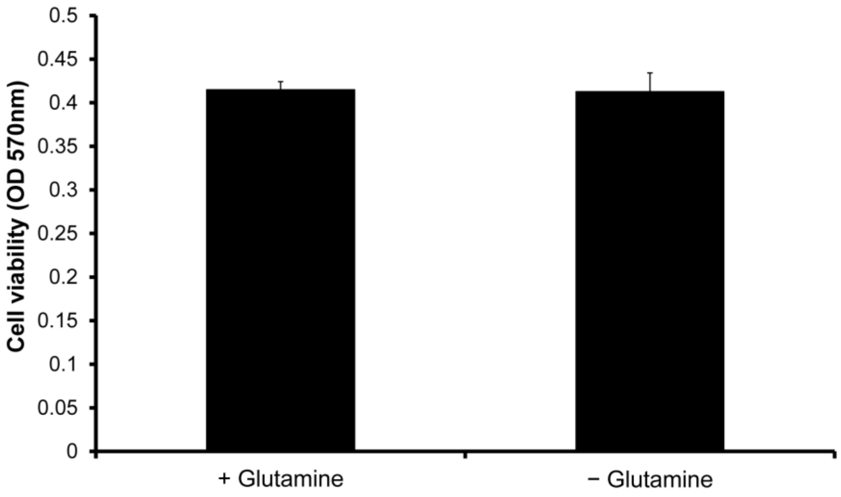

S3 Figure. Cell viability of AGS cells in the presence or absence of glutamine. AGS cells were cultured in glutamine-free medium or medium containing 2mM glutamine for 24 hours. Cell viability was measured by MTT assay.
