# Supplementary material for: Instrumental Role of Helicobacter pylori γ-Glutamyl Transpeptidase in VacA-Dependent Vacuolation in Gastric Epithelial Cells
Source: PLoS One. 2015 Jun 25;10(6):e0131460. doi: 10.1371/journal.pone.0131460 (PMC4482420; doi:10.1371/journal.pone.0131460)
Supplement: S4 Fig — Western blot of SDS-PAGE gels of H. pylon 88–3887 lysate with 10 MAbs and polyclonal anti-H. pylon GGT antibody. Lanes: 1, 1G5; 2, 1G10; 3, 1H5; 4, 2B5; 5, 2G1; 6, 4A11; 7, 1G1; 8, 3C10; 9, 3F4; 10, 4F11; 11, polyclonal anti-H. pylori GGT mouse IgG. Molecular weight markers are shown on the left. (PDF) [file pone.0131460.s004.pdf]

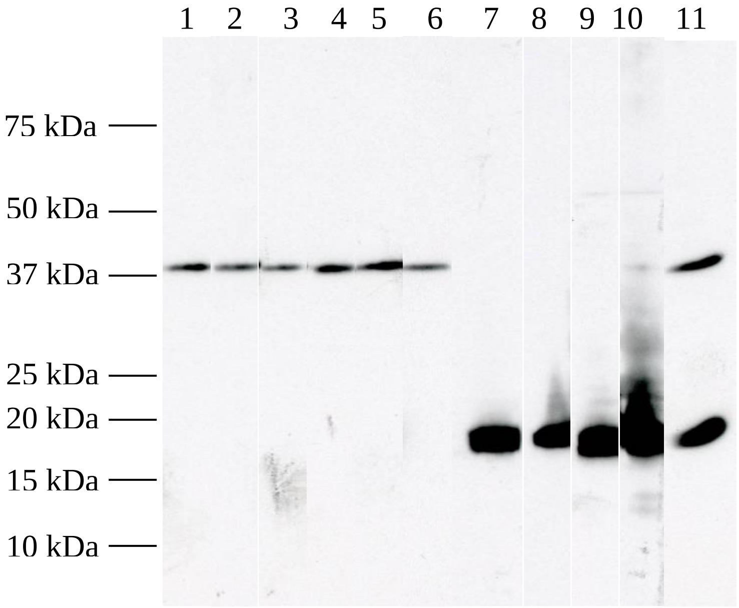

S4 Figure. Specificity of MAbs raised against rGGT. Western blot of SDS-PAGE gels of *H. pylon* 88-3887 lysate with 10 MAbs and polyclonal anti-*H. pylori* GGT antibody. Lanes: 1, 1G5; 2, 1G10; 3, 1H5; 4, 2B5; 5, 2G1; 6, 4A11; 7, 1G1; 8, 3C10; 9, 3F4; 10, 4F11; 11, polyclonal anti-*H. pylori* GGT mouse IgG. Molecular weight markers are shown on the left.
