# Supplementary material for: Instrumental Role of Helicobacter pylori γ-Glutamyl Transpeptidase in VacA-Dependent Vacuolation in Gastric Epithelial Cells
Source: PLoS One. 2015 Jun 25;10(6):e0131460. doi: 10.1371/journal.pone.0131460 (PMC4482420; doi:10.1371/journal.pone.0131460)
Supplement: S5 Fig — AGS cells were incubated with H. pylori WT, ggt-isogenic mutant or rGGT for 24 hours. Uninfected cells (UN) were included as the control. Results are represented as fold difference with respect to uninfected cells (taken as 1). *P<0.05. (PDF) [file pone.0131460.s005.pdf]

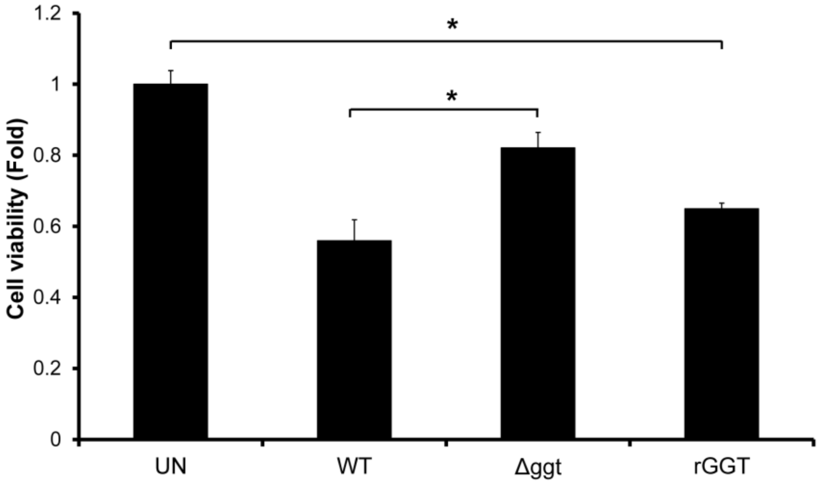

S5 Figure. Cell viability of AGS cells after co-culture with *H. pylori* or rGGT. AGS cells were incubated with *H. pylori* WT, *ggt*-isogenic mutant or rGGT for 24 hours. Uninfected cells (UN) were included as the control. Cell viability was measured by MTT assay. Results are represented as fold difference with respect to uninfected cells (taken as 1). **P*<0.05.
