# Supplementary material for: Instrumental Role of Helicobacter pylori γ-Glutamyl Transpeptidase in VacA-Dependent Vacuolation in Gastric Epithelial Cells
Source: PLoS One. 2015 Jun 25;10(6):e0131460. doi: 10.1371/journal.pone.0131460 (PMC4482420; doi:10.1371/journal.pone.0131460)
Supplement: S6 Fig — (A) AGS and (B) MKN28 cells were co-cultured with various H. pylori strains and their respective ggt-isogenic mutants as indicated for 24 hours (MOI 1:100). Two standard H. pylori strains (88–3887 and 26695) and four clinical strains (789, 840, 1034, 1018 were tested. The cells were subjected to neutral red uptake assay. Uninfected cells (UN) served as control. The clinical strains are all Type I strains with vacA s1 genotype and were isolated from gastric biopsies obtained from the gastric antrum within 2 cm of the pylorus in patients who underwent upper gastrointestinal endoscopy at the National University Hospital, Singapore. Strains 789 and 1018 were isolated from patients with gastric ulcer while strains 840 and 1034 were isolated from patients with non-ulcer dyspepsia. Experiments were performed in triplicates and values represent the means ± SD from 3 independent experiments. *P<0.05. (PDF) [file pone.0131460.s006.pdf]

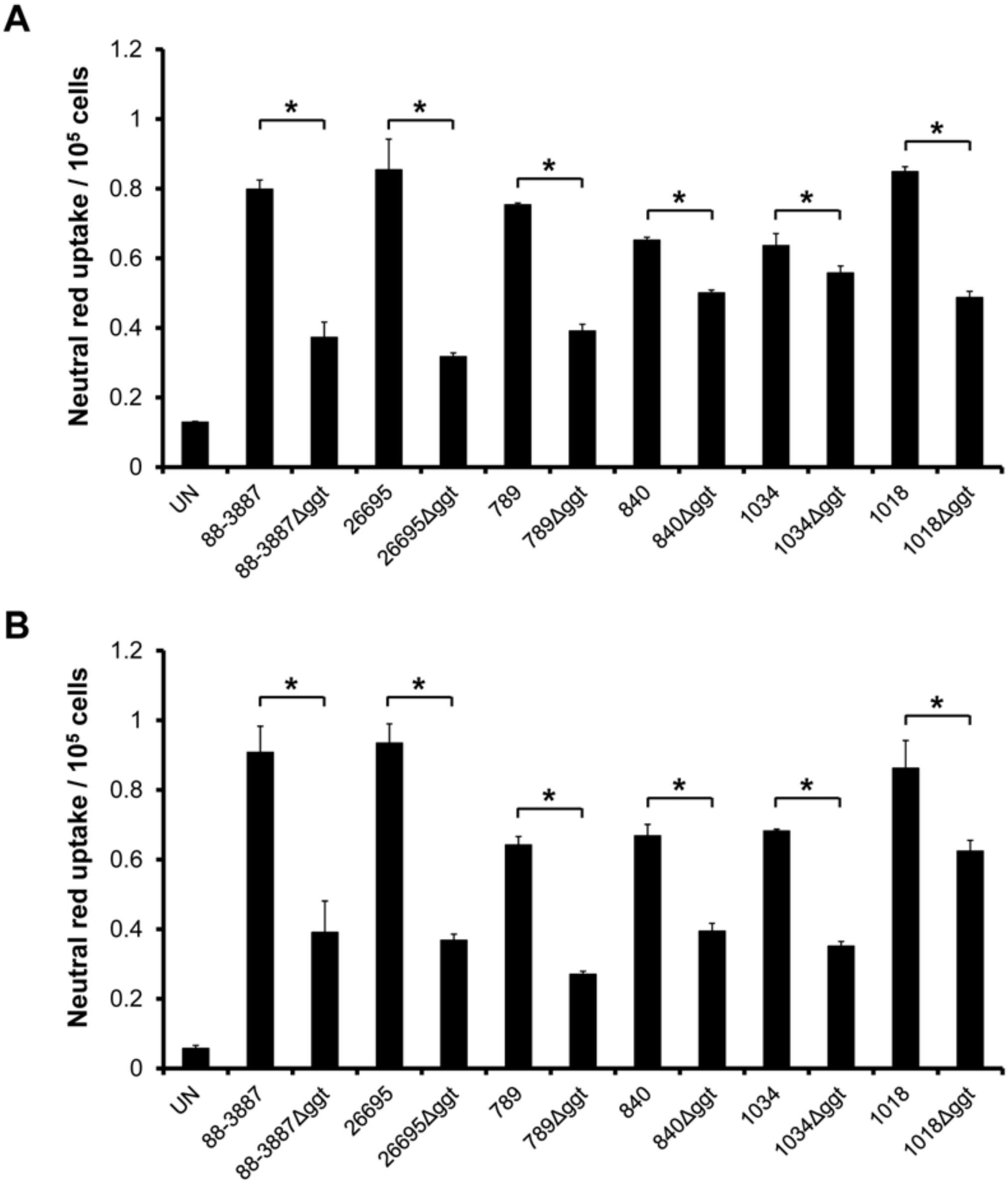

S6 Figure. Potentiation of vacuolation by *H. pylori* GGT is not strain-dependent nor cell-line dependent. (A) AGS and (B) MKN28 cells were co-cultured with various *H. pylori* strains and their respective *ggt*-isogenic mutants as indicated for 24 hours (MOI 1:100). Two standard *H. pylori* strains (88-3887 and 26695) and four clinical strains (789, 840, 1034, 1018 were tested. The cells were subjected to neutral red uptake assay. Uninfected cells (UN) served as control. The clinical strains are all Type I strains with *vacA* s1 genotype and were isolated from gastric biopsies obtained from the gastric antrum within 2 cm of the pylorus in patients who underwent upper gastrointestinal endoscopy at the National University Hospital, Singapore. Strains 789 and 1018 were isolated from patients with gastric ulcer while strains 840 and 1034 were isolated from patients with non-ulcer dyspepsia. Experiments were performed in triplicates and values represent the means ± SD from 3 independent experiments. *P<0.05.
