# Supplementary material for: Instrumental Role of Helicobacter pylori γ-Glutamyl Transpeptidase in VacA-Dependent Vacuolation in Gastric Epithelial Cells
Source: PLoS One. 2015 Jun 25;10(6):e0131460. doi: 10.1371/journal.pone.0131460 (PMC4482420; doi:10.1371/journal.pone.0131460)
Supplement: S7 Fig — Western blot analysis of H. pylori lysates probed using antibody against VacA. Lane 1, H. pylori WT lysate; Lane 2, Δggt lysate; Lane 3, ΔvacA lysate. (PDF) [file pone.0131460.s007.pdf]

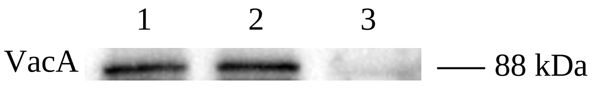

S7 Figure. Deletion of *ggt* gene does not affect the expression of VacA. Western blot analysis of *H. pylori* lysates probed using antibody against VacA. Lane 1, *H. pylori* WT lysate; Lane 2, Δ*ggt* lysate; Lane 3, Δ*vacA* lysate.
