# Supplementary material for: Instrumental Role of Helicobacter pylori γ-Glutamyl Transpeptidase in VacA-Dependent Vacuolation in Gastric Epithelial Cells
Source: PLoS One. 2015 Jun 25;10(6):e0131460. doi: 10.1371/journal.pone.0131460 (PMC4482420; doi:10.1371/journal.pone.0131460)
Supplement: S1 Table — (PDF) [file pone.0131460.s008.pdf]

S1 Table. Primers used for construction of various mutants of *H. pylori*.

Primer	Nucleotide sequence (5’→3’)	Reference
vacAF	CGTTGAGCGTTTTAGAAAGCAT	This study
vacACamR	CCCAGTTTGTCGCACTGATAAGCACAAAG<br>GGTG CGACTTTA	This study
vacACamF	ATCCACTTTTCAATCTATATCGCATACAC<br>CACAAGC TTGTTAT	This study
vacAR	CCCAAGTGGAATATTATGCGTT	This study
ureABR2	TCCCTAAAGGGATTTTCAAGATGT	[1]
ureCamF2	CCCAGTTTGTCGCACTGATAACCATGTGT<br>TCGT GGATGGCAA	[1]
ureCamR1	ATCCACTTTTCAATCTATATCATTCTCCTA<br>TTC TTAAAGTGTTTT	[1]
ureABF1	CATGGGGGCGTGGTGGATTA	[1]
CamR	TTATCAGTGCGACAAACTGGG	[1]
CamF	GATATAGATTGAAAAGTGGAT	[1]

Overlaps between primers vacACamR and CamF, primers vacACamF and CamR, primers ureCamF2 and CamR, primers ureCamR1 and CamF are underlined.

REFERENCES

1. Tan S, Berg DE. Motility of urease-deficient derivatives of Helicobacter pylori. J Bacteriol **2004**; 186:885-8.
