# Supplementary material for: Instrumental Role of Helicobacter pylori γ-Glutamyl Transpeptidase in VacA-Dependent Vacuolation in Gastric Epithelial Cells
Source: PLoS One. 2015 Jun 25;10(6):e0131460. doi: 10.1371/journal.pone.0131460 (PMC4482420; doi:10.1371/journal.pone.0131460)
Supplement: S2 Table — (PDF) [file pone.0131460.s009.pdf]

S2 Table. Summary of MAb isotypes and specificities.

Clone ID	Isotype	Specificity	
		Large subunit	Small subunit
1G5	IgG_1	+	-
1G10	IgG_1	+	-
1H5	IgG_1	+	-
2B5	IgG_1	+	-
2G1	IgG_1	+	-
4A11	IgG_1	+	-
1G1	IgG_{2b}	-	+
3C10	IgG_{2a}	-	+
3F4	IgG_{2a}	-	+
4F11	IgG_{2a}	-	+
